# The Association Between Smartphone Overuse and Cognitive Impairment Among Adults in the United Arab Emirates: A Cross-Sectional Study

**DOI:** 10.7759/cureus.99931

**Published:** 2025-12-23

**Authors:** Nour Mustafa, Abed Alhamid Alrais, Mohammad Alshammari, Aiya Horeie, Nouf Al Ali, Shamsa Almaazmi, Deepika Kamath M, Amal Hussein

**Affiliations:** 1 Basic Medical Sciences Department, College of Medicine, University of Sharjah, Sharjah, ARE; 2 Family and Community Medicine and Behavioral Sciences Department, College of Medicine, University of Sharjah, Sharjah, ARE

**Keywords:** ad8, cognitive impairment, dementia, digital health, mental health, sas-sv, smartphone, uae

## Abstract

Background

The widespread usage of smartphones has led to increasing concern regarding their potential adverse effects on cognitive health. While international studies have explored associations between excessive smartphone use and cognitive decline, there remains a gap in the literature specific to the United Arab Emirates (UAE).

Aim

This study aimed to investigate the association between smartphone overuse and cognitive impairment among adults in the UAE.

Methods

A cross-sectional study was conducted with a convenience sample of 401 adults aged ≥18 years across the UAE. Data were collected using an online self-administered questionnaire. Participants were categorized into three categories: low-risk, high-risk, and addicts based on the Smartphone Addiction Scale-Short Version (SAS-SV). The assessment of cognitive impairment was done through the Ascertain Dementia-8 Scale (AD8).

Results

A total of 281 participants (70%) were female, and 284 (71%) were in the age range of 18-24. Among the participants, 302 (75.3%) were classified as addicts, 75 (18.7%) were high-risk, and 24 (6%) were low-risk. According to the AD8 scale, the mean score was 1.9, and 182 (45.4%) of the participants were more likely to have cognitive impairment. A statistically significant association was observed between smartphone addiction and cognitive impairment (p = 0.002). The prevalence of cognitive impairment in addicts, high-risk, and low-risk subjects was 152 (50.3%), 22 (29.3%), and eight (33.3%), respectively. Smartphone overuse was also significantly associated with sleep disturbances (p < 0.001), headaches (p = 0.007), painful fingers (p = 0.015), and visual strain (p = 0.030). Depression was found to be significantly associated with cognitive impairment (p = 0.007).

Conclusion

The findings highlight a significant association between excessive smartphone use and cognitive impairment among adults in the UAE. These results emphasize the need for awareness campaigns, early screening initiatives, and further longitudinal research to explore causality and mitigate the cognitive risks associated with digital overuse.

## Introduction

Smartphones are now an integral part of our daily lives, widely used for communication, work, entertainment, and education. Despite their many benefits, concerns are growing about the potential impact of excessive use on mental and cognitive health. International research has suggested a connection between smartphone overuse and early signs of cognitive decline, including dementia-like symptoms. However, no studies have specifically explored this link in the UAE, where smartphone usage is particularly high.

As reported by Moshel et al., excessive screen use has been shown to impair cognitive performance, with attention being the most significantly affected domain, followed by deficits in executive functions [1]. A study conducted among university students has shown that those who overuse smartphones tend to demonstrate poorer cognitive performance, along with increased fatigue, more frequent eye-related symptoms, and greater discomfort in their dominant hand [2].

According to the Telecommunications and Digital Government Regulatory Authority (TDRA), smartphone penetration in the UAE is over 99% [3]. Recent studies indicate that a large proportion of UAE university students spend over seven hours online each day, with 84% of them using their smartphones for the majority of this time [4]. This trend of excessive smartphone use is concerning, as it has been associated with a range of adverse health outcomes, including increased anxiety, depressive symptoms, and disrupted sleep patterns.

According to the WHO's Dementia Fact Sheet, in 2021, 57 million people had dementia worldwide, and every year, there are nearly 10 million new cases [5]. However, specific data on the prevalence of dementia among adults in the UAE is limited.

This study aimed to investigate the association between smartphone overuse and early cognitive decline among adults in the UAE. Given that screen time in the UAE is significantly high, it is important to assess how prolonged use may affect brain function. The study will use validated scales to measure smartphone overuse and cognitive tests to evaluate areas such as attention and memory. It will also consider the physical effects of excessive screen exposure and whether there is a correlation between depression, smartphone overuse, and cognitive impairment. Gaining insight into these factors could help raise awareness about the risks of overuse and encourage further research on how digital habits influence long-term brain and psychological health.

## Materials and methods

Study design and setting

A cross-sectional study with a descriptive and analytic structure was conducted to assess the association between smartphone overuse and cognitive impairment among adults residing in the UAE. Data collection took place during the 2022-2023 academic year using an online survey platform. The questionnaire was distributed across social media platforms, including WhatsApp (Meta Platforms, Inc., Menlo Park, CA), Instagram (Meta Platforms Inc., Menlo Park, CA), and others, to reach a broad and diverse adult population across various Emirates.

Participants and procedure

A convenience sample of adults (N = 401; age ≥ 18) living in the UAE was selected for this study. Eligibility criteria included UAE residency and a basic understanding of either Arabic or English. Individuals previously diagnosed with dementia, or those who had suffered from brain tumors, head injuries, or strokes, were excluded due to potential overlap in symptoms that could confound the results. Participants were recruited online, and responses were collected via a self-administered Google Form questionnaire, estimated to take 10-12 minutes to complete.

Sampling size was determined using the formula:

$\mathrm{n} = \frac{4p(1 - p)}{\mathrm{SE}^2}$

Assuming a 50% expected prevalence (p = 0.5) and a 5% margin of error (SE = 0.05), the minimum sample size required was calculated to be 400. The choice of 50% was based on variability in previous literature and to ensure a sufficiently large sample for detecting associations within the general adult population, as opposed to elderly populations targeted in prior studies.

Sociodemographic variables

Participants reported on gender, age, nationality, marital status, educational level, employment status, monthly family income, living arrangement, smoking status, physical activity level, long-term health conditions (for instance, diabetes, hypertension, etc.), and mental health history. These items formed the first section of the questionnaire and were used to characterize the sample and control for potential confounding variables.

Smartphone overuse

Smartphone overuse was assessed using the Smartphone Addiction Scale - Short Version (SAS-SV) [6]. The scale consists of 10 items (e.g., “I am missing planned work due to smartphone use”) rated on a six-point Likert scale from one (strongly disagree) to six (strongly agree). The total score ranges from 10 to 60, with higher scores indicating greater smartphone addiction. The participants were categorized into "addicts", "high risk", and "low risk" groups according to their scores. For males, a score above 31 indicates addiction, while scores between 22 and 31 suggest a high risk of addiction, and scores less than 22 indicate low risk. For females, addiction is defined by a score exceeding 33, with scores ranging from 22 to 33 indicating a high risk, and scores less than 22 indicating low risk. Cut-off scores for defining smartphone addiction were obtained from the original Korean SAS-SV validation by Kwon et al. [6], whereas the score ranges used to classify participants as high-risk were based on the Spanish adaptation developed by Lopez-Fernandez [7]. The SAS-SV is a validated tool with established psychometric properties and was supplemented with questions on smartphone usage patterns and related health effects (e.g., sleep disturbance, visual issues).

Cognitive impairment assessment

Cognitive impairment symptoms were measured using the Ascertain Dementia 8 (AD8) Scale, an eight-item screening tool developed to detect early cognitive changes with a sensitivity of 74-85% and a specificity of 86% [8]. Each item assesses change in cognitive function (e.g., “I have trouble remembering appointments”), with responses categorized as “Yes, a change”, “No, no change”, or “Don’t know”. Participants were categorized into two groups: "normal cognition" and "cognitive impairment is likely to be present", according to their scores. A score of two or more suggests likely cognitive impairment. Additional items assessed participants’ awareness and knowledge about dementia, their confidence in caring for individuals with dementia, and prior participation in related events or conferences.

Data collection and ethical approval

The survey was developed by the research team in collaboration with supervisors, and its structure followed a three-part layout: Demographics, Smartphone Use, and Dementia Awareness and Screening. Instruments, including SAS-SV and AD8, were adopted from published sources. Though not pilot-tested, content validity was established via group discussion and expert supervision. Participation was anonymous and voluntary, with an information sheet and digital informed consent included at the beginning of the form. Ethical oversight was provided by the Human Research Ethics Committee at the University of Sharjah (REC number: RE-23-02-18-23-S).

Data analysis

Data were coded, entered, and analyzed using SPSS version 22 (IBM Corp., Armonk, NY). As appropriate to the type of data analyses, for univariate analyses, descriptive statistics, including measures to condense data (such as frequency and relative frequency), measures of central tendency (including mean, median, and mode), and measures of variability (standard deviation) were used. Bivariate analysis was conducted to study the relationship between variables. Inferential statistics tests, including chi-square and Mann-Whitney, were used as appropriate to the type of variables involved. A p-value < 0.05 was considered significant.

## Results

Smartphone use

**Figure 1:**
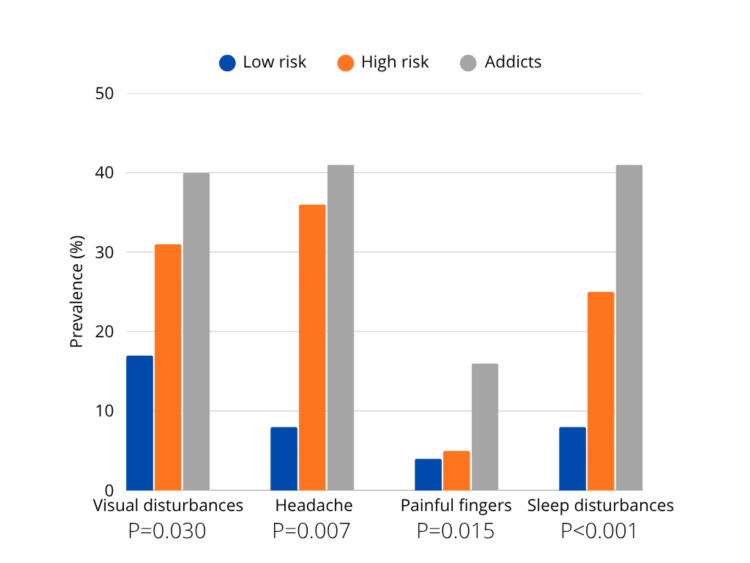
Smartphone overuse and adverse effects

A total of 268 participants (67%) used their smartphones for more than four hours daily, 96 (24%) for three to four hours, and 34 (9%) for less than two hours. Based on the SAS-SV scale, the mean score was 39.37. Additionally, 302 (75.3%) of the individuals were categorized as addicts, 75 (18.7%) were at high risk, and 24 (6%) were at low risk. Table 1 shows the association between demographics and smartphone overuse. Most importantly, nationality was a significant factor (p = 0.015), with the highest addiction rates seen among Emirati participants compared to other nationalities. Similarly, educational level had a significant association with smartphone overuse (p = 0.015), with those holding a Bachelor’s degree showing the highest addiction rates. Participants most frequently reported using their smartphones for longer periods than intended, experiencing difficulty refraining from smartphone use, and repeatedly checking their devices to monitor social media interactions, as shown in Table 2. Figure 1 shows a significant correlation between smartphone overuse and various adverse effects, most notably sleep disturbances, followed by headaches, finger pain, and vision problems.

**Table 1:** Demographics and smartphone overuse

Demographics		n (%)	Low-risk	High-risk	Addicts	p-values	Chi-square value
Gender	Male	120 (29.9)	6 (5.0)	22 (18.3)	92 (76.7)	0.847	0.332
	Female	281 (70.1)	18 (6.4)	53 (18.9)	210 (74.7)		
Age groups, years	18-24	284 (70.8)	16 (5.6)	60 (21.1)	208 (73.2)	0.270	5.174
	25-39	50 (12.5)	2 (4.0)	6 (12.0)	42 (84.0)		
	40+	67 (16.7)	6 (9.0)	9 (13.4)	52 (77.6)		
Nationality	Local	111 (27.7)	2 (1.8)	20 (18.0)	89 (80.2)	0.015	12.347
	Arab	260 (64.8)	18 (6.9)	45 (17.3)	197 (75.8)		
	Non-Arab	30 (7.5)	4 (13.3)	10 (33.3)	16 (53.3)		
Marital status	Single	295 (73.6)	19 (6.4)	63 (21.4)	213 (72.2)	0.298	7.250
	Married	96 (23.9)	4 (4.2)	11 (11.5)	81 (84.4)		
	Widowed	2 (0.5)	0 (0.0)	0 (0.0)	2 (100.0)		
	Divorced	8 (2.0)	1 (12.5)	1 (12.5)	6 (75.0)		
Educational level	High school and below	208 (51.9)	10 (4.8)	56 (26.9)	142 (68.3)	< 0.001	23.276
	Bachelor’s degree	154 (38.4)	12 (7.8)	11 (7.1)	131 (85.1)		
	Post-graduate degree	39 (9.7)	2 (5.1)	8 (20.5)	29 (74.4)		
Employment Status	Employed	74 (18.5)	6 (8.1)	9 (12.2)	59 (79.7)	0.507	5.293
	Unemployed	55 (13.7)	5 (9.1)	9 (16.4)	41 (74.5)		
	Retired	6 (1.5)	0 (0.0)	1 (16.7)	5 (83.3)		
	Student	266 (66.3)	13 (4.9)	56 (21.1)	197 (74.1)		
Residing status	Living alone	85 (21.2)	4 (4.7)	10 (11.8)	71 (83.5)	0.133	4.039
	Living with family	316 (78.8)	20 (6.3)	65 (20.6)	231 (73.1)		
Total		401 (100.0)	24 (6.0)	75 (18.7)	302 (75.3)		

**Table 2:** Smartphone Addiction Scale - Short Version (SAS-SV) items

Items	Strongly agree/agree	Weakly agree	Weakly disagree	Strongly disagree/disagree
Missing planned work due to smartphone use	168 (41.9)	97 (24.2)	41 (10.2)	95 (23.7)
Difficulty concentrating due to smartphone use	171 (42.6)	101 (25.2)	46 (11.5)	83 (20.7)
Experiencing pain in wrists or neck while using a smartphone	137 (34.2)	89 (22.2)	52 (13.0)	123 (30.7)
Inability to stand not having a smartphone	237 (59.1)	78 (19.5)	34 (8.5)	52 (13.0)
Feeling impatient or fretful when not holding a smartphone	160 (39.9)	89 (22.2)	63 (15.7)	89 (22.2)
Thinking about the smartphone even when not using it	128 (31.9)	88 (21.9)	77 (19.2)	108 (26.9)
Continued use despite negative impacts on daily life	149 (37.2)	95 (23.7)	61 (15.2)	96 (23.9)
Constantly checking the smartphone to follow social media conversations	201 (50.1)	90 (22.4)	46 (11.5)	64 (16.0)
Using the smartphone longer than intended	247 (61.6)	84 (20.9)	32 (8.0)	38 (9.5)
Being told by others that smartphone use is excessive	107 (26.7)	91 (22.7)	73 (18.2)	130 (32.4)

Cognitive impairment

**Figure 2:**
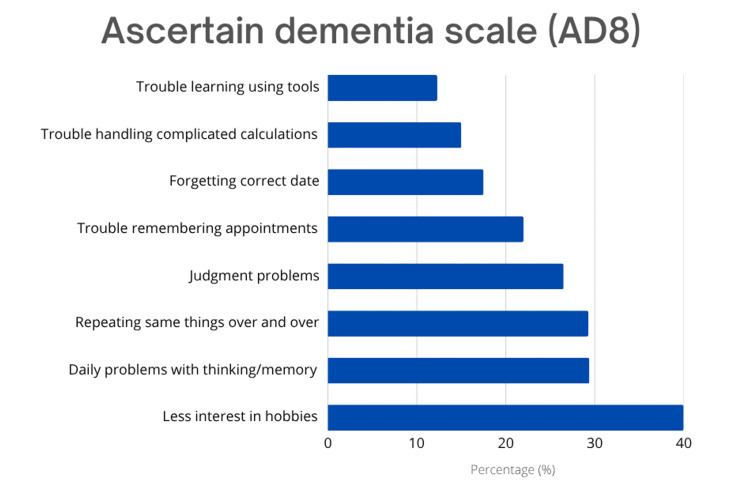
Ascertain Dementia-8 Scale (AD8) scale

Based on the AD8 scale, the mean score was 1.9, and 219 (54.6%) of the participants demonstrated normal cognitive function, while 182 (45.4%) were likely to have cognitive impairment. Table 3 shows no significant relationship between age and cognitive decline. However, a significant correlation was found between depression and cognitive impairment (p = 0.007), with 31 (63.3%) of those diagnosed with depression demonstrating a higher likelihood of cognitive issues. The most frequently reported symptom on the AD8 scale was reduced interest in hobbies, followed by difficulties with daily thinking and memory, as shown in Figure 2.

**Table 3:** Variables and cognitive impairment

Variables		Normal cognition	Cognitive impairment is likely to be present	p-values	Chi-square value
Gender	Male	69 (57.5)	51 (42.5)	0.448	0.576
	Female	150 (53.4)	131 (46.6)		
Age groups, years	18-24	155 (54.6)	129 (45.4)	0.677	0.779
	25-39	25 (50.0)	25 (50.0)		
	40+	39 (58.2)	28 (41.8)		
Nationality	Emirati	64 (57.5)	47 (42.3)	0.751	0.574
	Arab	139 (53.5)	121 (46.5)		
	Non-Arab	16 (53.3)	14 (46.7)		
Marital status	Single	164 (55.6)	131 (44.4)	0.513	2.297
	Married	48 (50.0)	48 (50.0)		
	Widowed	1 (50.0)	1 (50.0)		
	Divorced	6 (75.0)	2 (25.0)		
Educational level	High school and below	116 (55.8)	92 (44.2)	0.653	0.853
	Bachelor’s degree	80 (51.9)	74 (48.1)		
	Post-graduate	23 (59.0)	16 (41.0)		
Employment status	Employed	37 (50.0)	37 (50.0)	0.507	1.277
	Unemployed	29 (52.7)	26 (47.3)		
	Retired	4 (66.7)	2 (33.3)		
	Student	149 (56.0)	117 (44.0)		
Residing status	Living alone	39 (45.9)	46 (54.1)	0.069	3.317
	Living with family	180 (57.0)	136 (43.0)		
Depression diagnosis	Yes	18 (36.7)	31 (63.3)	0.007	7.199
	No	201 (57.1)	151 (42.9)		
Total		219 (54.6)	182 (45.4)		

Cognitive impairment and smartphone overuse

**Figure 3:**
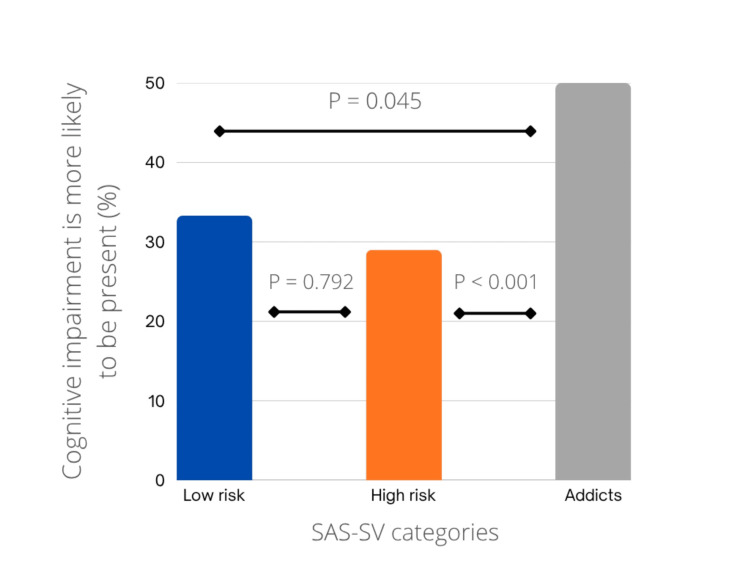
Cognitive impairment and smartphone overuse

A significant association was observed between cognitive impairment and smartphone usage (p = 0.002, chi-square value = 12.183). Figure 3 shows that cognitive impairment was more prevalent among 152 (50.3%) of the individuals classified as addicts, compared to 22 (29.3%) of those at high risk and 8 (33.3%) of those at low risk. The Mann-Whitney U test was employed to determine which specific groups showed a significant difference. The findings revealed a higher significance between the addict and high-risk groups (p < 0.001, Mann-Whitney U score = 8037.0), followed by the addict and low-risk groups (p = 0.045, Mann-Whitney U score = 2754.0). However, no significant difference was found between the high-risk and low-risk groups (p = 0.792, Mann-Whitney U score = 870.0).

## Discussion

The results of the research indicate a statistically significant association between the excessive use of smartphones and a risk of cognitive impairment, as measured with the AD8 scale. To our knowledge, there have been no previous studies conducted in the UAE that show the correlation between smartphone overuse and cognitive impairment. Prior studies have supported our findings, as reported by Neophytou et al. [9], excessive smartphone and screen use has been linked to cognitive, behavioral, and emotional difficulties in adolescents and young adults, and may also increase vulnerability to neurodegenerative conditions such as dementia later in life. A systematic review found that excessive smartphone use is associated with changes in attention span, memory retrieval, and executive functioning [10]. A cross-sectional study conducted among 251 Saudi adults found a significant association between excessive smartphone use and cognitive impairment [11]. Excessive digital device use has also been linked to neuropsychological effects, including disruptions in brain network connectivity that are important for cognitive control and higher-order processing [12]. The high coefficient of this association in our study, both verified by chi-square and Mann-Whitney measures, lends very strong empirical support to these results.

Recent research continues to highlight the impact of screen media activity on brain structure in youth. A 2024 study utilizing data from the Adolescent Brain Cognitive Development (ABCD) Study found that excessive screen time was associated with structural variations in brain regions involved in cognitive and emotional processing, including the thalamus, prefrontal cortex, and brainstem. These changes were also linked to sleep problems and the development of psychopathological symptoms over time, suggesting that screen media activity may influence both brain maturation and mental health outcomes [13]. These findings underscore the importance of monitoring screen use in children and adolescents to mitigate potential neurodevelopmental and psychological consequences.

Moreover, this study showed important associations between smartphone overuse and somatic complaints, including headaches, visual disorders, painful fingers, and sleep disturbances. Recent reviews indicate that excessive smartphone use in migraine patients is associated with longer and more frequent headaches. It also negatively impacts sleep, leading to poorer sleep quality and increased daytime sleepiness, which in turn reduces overall quality of life [14]. In addition, prolonged smartphone use may cause ocular strain and other visual discomforts, highlighting the importance of moderating usage to prevent postural and visual issues [15].

Contrary to conventional expectations, the age factor did not contribute significantly to cognitive impairment in this sample. Even though dementia may be prominently related to the elderly, based on our data, youngsters as young as 18-24 years old, having the most participants, also had indications of cognitive deterioration.

The study supports a significant association between depression and cognitive impairment. A systematic review and meta-analysis found that individuals with depression show moderate deficits in executive function and attention, along with smaller deficits in memory, suggesting that cognitive impairment is a core feature of depression rather than a secondary symptom [16].

The absence of a significant relationship between smartphone overuse and depression in this study contrasts with previous research, which suggested that online social support and digital connectedness may help alleviate stress and emotional distress in the short term [17]. Nevertheless, further research is needed to investigate the underlying motives for smartphone use and the role of emotional dependence in shaping long-term mental health outcomes.

The present research also gives room for other research in the future. The neuroimaging and neuroimaging biomarker approach has an urgent niche in examining the neurological basis of smartphone overuse. In addition, we should consider patterns of digital behavior, including the type of use (passive scrolling vs. active communication), app engagement, screen time before bedtime, and so forth, that may help us to distinguish precisely which kind of behavior is most harmful to intellectual processes. Furthermore, the use of comprehensive neuropsychological instruments and clinician-administered cognitive assessments, rather than relying solely on self-administered questionnaires, would strengthen the accuracy and validity of findings in this field.

Screening for smartphone overuse can be incorporated into clinical practice, particularly when patients present with symptoms that may be associated with excessive smartphone use, such as headaches, sleep disturbances, concentration difficulties, visual strain, or musculoskeletal complaints. Using validated tools like the SAS-SV allows clinicians to identify individuals at risk and address problematic use early. Integrating such screening into routine assessments may help guide appropriate counseling, behavioral interventions, and further evaluation when needed.

Limitations

Although the present study contributes valuable insights into the relationship between smartphone overuse and cognitive decline among young adults, several limitations should be acknowledged. The relatively small sample size, use of convenience sampling, and the predominance of young participants limit the generalizability of the findings to other demographic groups. In addition, self-selection bias may have occurred, as individuals who chose to participate might differ from those who did not, particularly in their awareness of or interest in smartphone-related behaviors. Reliance on self-reported measures may also introduce bias in estimating both smartphone use and cognitive symptoms. Another limitation pertains to the gender imbalance within the sample, as the proportion of female participants was notably higher than that of males, potentially affecting the representativeness of the results.

Moreover, the study did not distinguish between occupational and non-occupational smartphone use, an important factor given that many young adults, such as influencers or content creators, rely on smartphones for work, which may limit the ecological validity of the findings. Finally, the cross-sectional design precludes causal inference; while a significant association was observed between smartphone overuse and cognitive impairment, the directionality of this relationship remains uncertain.

In addition, our results challenge a portion of the conflicting data available in contemporary literature. For instance, Qi et al. [18] concluded that the odds of cognitive impairment are reduced in smartphone users compared to non-users. These discrepancies could be due to the methodological differences and the target population.

## Conclusions

The study revealed a significant association between excessive smartphone use and a higher risk of cognitive impairment among adults in the UAE. A total of 182 participants (45.40%) showed signs of cognitive decline, with a greater prevalence among those identified as smartphone addicts. Additionally, smartphone overuse was linked to several physical symptoms, which may further contribute to cognitive difficulties.

This research is the first to examine this relationship within the UAE, adding valuable regional insight to the limited existing literature. However, certain limitations should be noted, including the use of convenience sampling, reliance on self-reported data, and a predominantly young sample, which restricts generalizability. Given its cross-sectional design, causality cannot be established. Raising public awareness about the cognitive risks of smartphone overuse and promoting healthier usage habits are recommended. Future studies should employ more rigorous designs and explore the neurobiological mechanisms underlying the link between smartphone use and cognitive impairment.
